# The BeHealthyR Study: a randomized trial of a multicomponent intervention to reduce stress, smoking and improve financial health of low-income residents in Rotterdam

**DOI:** 10.1186/s12889-018-5728-7

**Published:** 2018-07-18

**Authors:** Sara S. Shagiwal, Astrid Schop-Etman, Iris Bergwerff, Wil Vrencken, Semiha Denktaş

**Affiliations:** 1 Department of Social and Behavioral Sciences, Erasmus University College/Erasmus University Rotterdam, Rotterdam, The Netherlands; 2 Indigo Rijnmond, Rotterdam, The Netherlands; 3 Avant Sanare, Rotterdam, The Netherlands

**Keywords:** Health disparities, Multicomponent intervention, Perceived health, Socioeconomic groups, Smoking, Stress

## Abstract

**Background:**

Compared to higher socioeconomic status (SES) groups, those in lower SES groups are financially strained, experience higher rates of smoking-related morbidity, are in poorer health and have reduced life expectancy. This is especially true for the city of Rotterdam, where a large inequality in health is observed between low and high SES groups. The BeHealthyR study (Dutch: Grip en Gezondheid) is a randomized controlled trial (RCT) which will evaluate the impact of a theory-based multicomponent behavior intervention aiming to reduce stress, smoking, and improve financial health by means of a group-based stress management program combining cognitive and behavioral techniques, and nudges in low-SES residents living in Rotterdam.

**Methods:**

The BeHealthyR study is a three-arm RCT. Between February 2018 and July 2019, low-SES participants who perceive stress, smoke, are financially strained and reside in Rotterdam (one of the four largest cities in The Netherlands) are recruited. Subsequently, participants are randomly assigned to either a stress management condition (SM), stress management with a buddy condition (SM-B) or a control condition (CC). Participants in the SM and SM-B conditions will attend four weekly group sessions (1.5 h/session) and a follow-up session eight weeks later. The SM condition includes psychoeducation and exercises, and cognitive and behavioral intervention techniques. Demographic data and objective measures will be collected at baseline (T0), four weeks post-baseline (T1), and twelve weeks post-baseline (T2). Primary outcome measures are to reduce stress, smoking and improve financial health. We hypothesize that low-SES participants in the intervention conditions, compared with those in the control condition, will experience less stress, smoke less and have improved financial health.

**Discussion:**

This study is a group-based intervention which aims to investigate the effects of a theory-based behavioral change intervention employing several components on reducing stress, smoking, and improving financial health in low-SES residents living in Rotterdam. If effective, the findings from the present study will serve to inform future directions of research and clinical practice with regard to behavioral change interventions for low-SES groups.

**Trial registration:**

ClinicalTrials.gov (ID: NCT03553979). Registered on January 1 2018.

**Electronic supplementary material:**

The online version of this article (10.1186/s12889-018-5728-7) contains supplementary material, which is available to authorized users.

## Background

Globally, smoking kills about seven million people each year and is the leading cause of preventable death and diseases including cancer, cardiovascular disease and respiratory disease [1]. In the Netherlands, 24.1% of the Dutch population aged 18 years and older smoked in 2016 [2]. It is estimated that more than 27,110 people die of smoking and smoking-related diseases each year [3]. Similar trends are observed in the four largest cities of the Netherlands (Amsterdam, The Hague, Rotterdam, and Utrecht). The most pronounced being Rotterdam [4]. Although the proportion of adult smokers in Rotterdam has decreased slightly since 2010, the difference in proportion of smokers across SES groups has increased [4]. Compared to those with high-SES, low-SES groups are more likely to smoke more, to suffer more from smoking-related diseases and are less likely to quit smoking. As a result of the unequal distribution of smoking across SES groups, smoking is now more of a problem among lower SES groups [4].

### Stress and smoking in low-SES groups

Smoking initiation, continuation, addiction and cessation among low-SES groups is a multidetermined behavior influenced by a combination of cultural (i.e. a constellation of attitudes, beliefs, perceptions, and personality), material (i.e. financial problems, material, and social deprivation), and psychosocial factors (i.e. stressors and coping styles) [5]. It has been posited that exposure to daily stressors play a critical role in smoking initiation, continuation, addiction, and cessation [5–8]. Low-SES individuals have been found in several studies to experience stressors (i.e unemployment, financial worries or poor health) more frequently and more acutely in their daily lives than their high-SES counterparts [9–11]. For these individuals, the stresses of situational constraints consume a great deal of their cognitive resources, attention, and may over time erode their psychosocial resources (i.e. coping strategies). This decrease in cognitive function and lack of psychosocial resources in return contribute to more stress, a higher mental burden (reduced mental capacity), cognitive bias (i.e. information-processing errors), and present bias (i.e. choosing present above future rewards) [12]. As a result of the ongoing stress, the impaired cognitive function and lack of psychosocial resources, low-SES individuals often attempt to ameliorate these effects by frequently enacting unhealthy behaviors which will give a temporary relief, such as resorting to alcohol consumption or smoking [13].

**Fig. 1:**
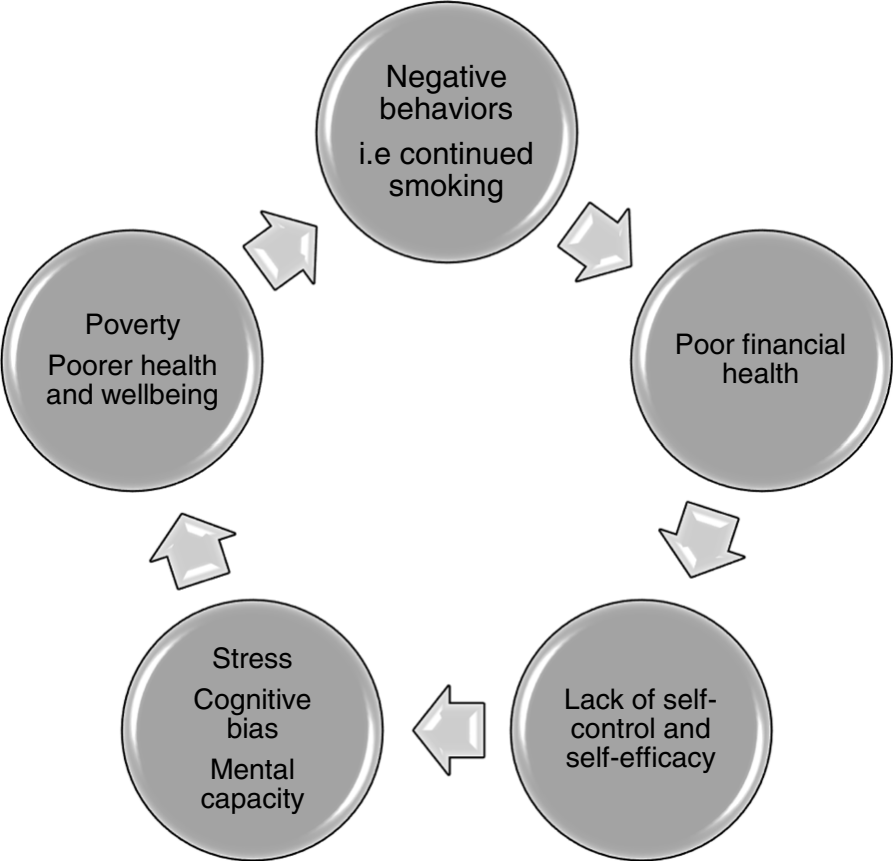
The viscous cycle of smoking on health and wellbeing

Despite the adoption of smoke-free policies (i.e. increasing cigarette costs and banning smoking in public areas), these policies may have had unintended consequences (i.e smoking outside banned areas) among low-SES groups who continue to smoke at higher rates [14]. Although low-SES groups are as motivated to quit smoking as their high-SES counterparts, they are less likely to succeed. Barriers for smoking cessation among low-SES groups have been identified and include higher levels of chronic stress [6, 7], pro-smoking community norms and higher nicotine dependence (due to smoking more cigarettes per day) [8, 15, 16]. Other barriers identified include financial and cultural barriers which limit access to cessation programs [8]. As a result of these barriers, low-SES groups are caught in a vicious cycle; the continued smoking drains the limited finances (i.e. difficulty paying for essentials due to money spent on cigarettes) which leads to lack of self-control and self-efficacy (confidence in the ability to bring around a change). The lack of self-control and self-efficacy leads to greater stress, reduced mental capacity and higher risk of cognitive biases which in turn leads to poorer health behavior (i.e maintaining smoking), poverty and poorer health and wellbeing (Fig. 1).

### Interventions targeting low-SES populations

While the effectiveness of behavioral change interventions (BCIs) has been implemented and evaluated for the general population, evidence on their effectiveness in low-SES groups is sparse and conflicting. Recent systematic reviews and meta-analyses conclude that, although there is some evidence of the benefits of BCIs for the general population, there is shortage of effective BCIs targeting low-SES groups [17–19]. There are several possible reasons why previous BCIs have been less effective. First, existing BCIs have generally been low- intensity consisting of providing advice and information only. Second, BCIs have lacked a solid theoretical foundation. Third, BCIs have focused on targeting a single risk behavior (i.e. smoking) instead of multiple risk behaviors. Research suggests that for BCIs to be effective, they should have a sound theoretical foundation allowing for appropriate determinants of behavior change to be targeted and effective behavior change techniques (BCTs) to be identified and used [20]. Further, previous research indicates that low-SES groups have more difficulties with cognitive orientation due to the high level of required homework assignments [21]. Therefore, for optimum effectiveness, BCIs targeting low-SES groups should be designed to utilize an experiential learning approach as this approach will likely lead to changes in attitude than traditional didactic instruction.

### Theoretical base of the BeHealthyR intervention

**Fig. 2:**
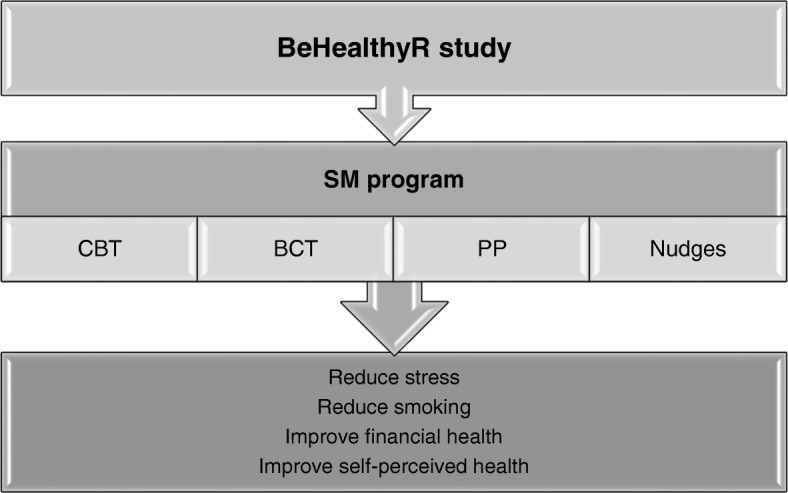
Conceptual model of BeHealthyR intervention. SM = stress management program; CBT = cognitive behavior therapy; BCT = behavioral change technique; PP = positive psychology

Figure 2 outlines the conceptual framework of the BeHealthyR intervention. This intervention is based on several theories approaching stress management (SM) techniques from a multifocal view starting with first raising awareness about the concept of stress and progressing to more complex cognitive activities (i.e. change negative stressor appraisals via cognitive restructuring and enhance coping skills) to behavioral activities (i.e. various relaxation techniques such as breathing techniques and progressive muscle relaxation). Having a theoretical framework helps to specify which variables affect the outcome of interest and gives the possibility to find connections between these variables. The evidence for each of the theory-based components of the current intervention is considered in turn below.

#### Lazarus’s transactional stress-coping model

Evidence shows that low-SES groups in general face a wide array of stressors than their high-SES counterparts. Stressors may include financial stress, unemployment or poor housing conditions. According to Lazarus’ transactional model of stress and coping (TMSC), stressors are defined as “demands made by both the internal and external environments that upset the balance of an individual, thus affecting both physical and psychological well-being and requiring action to restore balance” [22]. In order to manage the effects of stressors, three key consecutive processes are acknowledged: primary appraisal, secondary appraisal and coping strategies [23]. During the primary appraisal phase, once confronted with a stressor, one evaluates the consequences of the stressor on one’s wellbeing, for example, whether the stressor is harmful, threatening or irrelevant. If the stressor is considered harmful, one moves on to the secondary appraisal phase, during which one determines whether one has enough personal, social, and coping resources to get through the stressor with a desirable outcome [23, 24]. During both appraisal phases, if one experiences that one’s situational demands (i.e. risk, uncertainty, difficulty) outweighs one’s perceived resources (i.e. social support and expertise), one perceives negative stress response. This stress response, characterized by one’s personality characteristics (i.e. locus of control), history of stressors (i.e. loss of a loved one), coping resources and social support, involves different behavioral domains (i.e. physiological, cognitive, emotional). According to the TMSC, these domains are interrelated and thus a change in one will bring a change in the others [23]. Once one experiences negative stress, one moves on to using coping strategies to cope with the perceived stress. According to Lazarus, coping is defined as “constantly changing cognitive and behavioral efforts to manage external and/or internal demands that are appraised as taxing or exceeding the resources of a person” [23]. One can cope with stress using either: 1) emotion-focused coping (i.e. to regulate negative emotions that occur due to the stressful event); or 2) problem-oriented coping (i.e. to either resolve/change the stressful event or one’s behavior towards the stressful event). Also, a distinction between avoidance coping (one avoids the stressors) and active coping techniques (one confronts the stressors) have been discussed. Coping is determined by cognitive appraisal which is influenced by coping resources. Resources that are often considered to facilitate one’s coping efforts can be physical (i.e. health, energy), social (i.e. social support), psychological (i.e. beliefs, self-esteem, locus of control), or material (i.e. financial) [23].

Low-SES individuals have different ways of coping with a wide array of stressors, for example, by resorting to smoking [7]. Following the TMSC, how low-SES individuals perceive stressors and manage to cope with them determines the amount of the stress that is experienced. In the core component of the BeHealthyR intervention, a tailored SM course to meet the needs of low-SES individuals is integrated. In the SM course, low-SES individuals learn what stress is, how to identify and differentiate between types of stress, how to bolster their coping repertoire (intra-interpersonal skills) and how to apply the taught coping strategies in a more adaptive, functional or coping-orientated fashion. In summary, the SM course can be viewed as a tool to help low-SES individuals to first become aware of the impact of their maladaptive stress-engendering behaviors (i.e. avoidance, rumination, lack of social support) and second, to help them to change these maladaptive behaviors by bolstering their coping repertoire. Studies show that barriers to participation in BCIs by low-SES groups is due to financial costs, transportation and time [21]. To maximize participation retention in the current study, the SM course is offered free of charge and given in community centres in the participants’ neighborhoods in order to facilitate social cohesion among participants. Further the SM is given as a course rather than as therapy, so it is non-stigmatizing. This is important since these groups suffer from the stigma of being viewed as a societal burden, lazy and unmotivated [25].

#### Cognitive behavioral therapy

Cognitive behavioral therapy (CBT) is a psychosocial intervention that integrates the theoretical perspectives and therapeutic techniques of cognitive theory, which focuses on one’s perceptions and thoughts rather than behavior [26], and behavioral theory, which is based on the idea that a functional or dysfunctional behavior is learned and that behavior that is learned can be unlearned and replaced by a functional behavior [27]. CBT has its roots in a belief that one’s thoughts (cognitions), feelings (emotions) and behavior (response) are interconnected, and that changes in any one of the elements will bring a change in the others. CBT is based on the premise that maladaptive thoughts contribute to the maintenance of emotional distress and dysfunctional behaviors [28]. According to Beck’s cognitive model, maladaptive thoughts are largely caused by three mechanisms: cognitive bias (information-processing errors), negative self-schemas (negative beliefs/expectations about the self) and cognitive triad (negative thoughts about the self, the world and the future) [29]. Beck theorized that negative self-schemas develop in early life and remain latent until they are activated by a stressor. When a cognitively vulnerable person experiences a stressor, the person’s negative self-schema is activated. This activation triggers a cascade of activity that leads the person to interpret experiences in a distorted and negative way. These negative interpretations, in turn, lead to negative thoughts about the self, the world and the future (cognitive triad) which promote further negative emotions [30]. Beck developed interventions to change patterns of maladaptive thoughts and dysfunctional behavior and posited that these changes would change and improve negative emotions, respectively [30]. These changes can be achieved by cognitive restructuring, a main cognitive component of CBT which teaches one to dissect the relationships among one’s emotions, cognitions, and behaviors in order to identify and conceptualize one’s distorted thoughts and maladaptive behaviors in a more adaptive, functional or coping-orientated fashion [31]. Other components of CBT designed to alter one’s maladaptive thoughts include recognizing behavioral cues, identifying and developing strategies to avoid and cope with high-risk situations, building problem-solving skills and progressive relaxation [31]. CBT has been proven to be effective in the treatment of mental health problems (i.e. anxiety, panic and eating disorders) [28] and stress management [26]. However, the evidence base of the efficacy of CBT interventions in low-SES groups is limited [28].

Low-SES individuals have negative thought patterns and emotions and lack control over their lives. By integrating a CBT component, this study aims to teach low-SES participants to identify their negative thought processes, to replace them with more realistic appraisals and to endow them with a set of coping skills to deal with stress-inducing thoughts.

#### Positive psychology

Positive psychology (PP) initiated by Seligman in 1998, is defined as the positive development of a person as characterized by high self-efficacy (confidence in one’s ability to achieve a goal), optimism, hope (a positive motivational state) and resilience (adaption to a stressor) [32]. A main principle of PP is that psychologists should not ‘fix’ people’s problems, rather, it should help people to function optimally and improve their wellbeing [33, 34]. In the past few years, research has emphasized that some of the CBT commonalities overlap with some elements of research in positive psychology [33]. While CBT focuses on modifying maladaptive thoughts and dysfunctional behaviors, PP aims to repair the worst things in life but also build the best qualities in life [33]. Because of the conceptual overlap between CBT and PP, previous research has emphasized that the short and long-term efficacy of CBT can be enhanced by incorporating principals of PP, especially principals that are related to enhancing positivity and wellbeing [33]. The efficacy of multicomponent PPIs on improving well-being have been studied in several studies [35, 36]. Results from two meta-analyses show that PPIs can be effective in improving wellbeing [37, 38].

Given that low-SES groups are known to exhibit low self-efficacy, low optimism, less hope (about the self, the world and the future) and are less resilient when faced with a stressor, it is crucial to target these four cognitive components to improve their wellbeing. In the current study, a PP component is integrated using an experiential psychoeducational approach. Using this approach, topics on how to replace negative thoughts with positive thoughts, how to think optimistically (about the self, the world and the future) and how to increase resilience by learning and developing coping skills when faced with a stressor will be discussed during the group sessions.

#### Behavioral change techniques (BCTs)

##### Self-affirmation

Research shows that low-SES groups suffer from the stigma of being viewed as a societal burden, lazy and unmotivated [25]. Such stigma can activate the stress system which in turn can lead to poorer cognitive performance, reduce the working memory capacity and cause low-SES groups to forego beneficial programs [25]. A prominent theoretical advance in the interpretation of stigma is self-affirmation theory, which posits that people have a basic need to maintain self-worth and integrity, especially when it is threatened [39]. In the realm of identity-threatening events and information, encouraging an individual to self-affirm important values may help the individual to cope better with the treat, to have a broader perspective of the threat and to uncouple the self and threat, reducing the threat’s impact on the self [39]. In support of these ideas, self-affirmation research show greater acceptance of threatening health messages, has positive effects on cognitions like intention and self-efficacy, and promote behavior change regarding various health risks, such as smoking. For example, when affirmed, smokers are more open to accepting anti-smoking messages [40].

##### Goal-setting

The goal-setting theory states that goals affect motivation and behavior. A goal by definition is the aim of a person’s desire to achieve an action. According to Locke and Latham, goals affect performance through four mechanisms: direction, effort, persistence and strategy development [41]. Direction refers to how individuals direct their focus and effect toward goal-related activities. Effort is related to setting the goals. Setting high and challenging instead of easy ‘do your best’ goals has cognitive and motivation benefits because more effort is needed to achieve them. Similarly, persistence is related to effort. When an individual sets high and challenging goals, this individual will exert more effort and time to achieve these goals [41]. Goal-setting is also addressed in social cognitive theory (SCT) and cognitive behavior therapy (CBT), which emphasize the importance of setting achievable goals as a way to increase self-efficacy leading to behavior change [42]. Goal-setting strategies have been effective in promoting wellbeing [43, 44] and can effectively be used on any domain in which the individual or group has no control over health outcomes. This may especially be true for low-SES population as this population often experience lower self-control and lower self-efficacy to bring about a change in behavior [19].

#### Buddy-system

Social support refers to a social network’s provision of psychological and material resources intended to benefit an individual’s ability to cope with stress [45]. Lakey and Cohen presented an important theoretical perspective on the relationship between social support and health, namely the stress and coping perspective [46]. The stress and coping perspective, which draws from the TMSC model of Lazarus [23] proposes that the presence of social support buffers a person from the negative impact of stressors. In this view, perceived availability of social support may bolster’s one’s ability to cope with demands, further minimize the physiological responses to the stressful situation (i.e. increased heart rate) and facilitate healthful behaviors [47]. Social support has been applied in different contexts [48]. However, there is limited evidence about how well social support interventions work in low-SES groups. Previous reviews highlighted social support as a main active ingredient for promoting behavior change in low-SES populations [19, 49]. Evidence indicates that low-SES groups lack social support and self-control [5]. Therefore, by providing social support in the form of a buddy might benefit low-SES individuals. In this study, social support in the form of a buddy may: 1) reduce the impact of stressors in low-SES groups and help them cope better with a stressor (buffering effect); 2) increase sharing of health information (direct effect); 3) provide emotional support (i.e attempt to quit smoking); and 4) provide support on how to manage one’s finances.

#### Nudges

From past research it emerges that low-SES participants are often difficult to reach for preventive interventions and once they have been reached, they seem more likely to drop out [21]. To prevent drop-out and enhance participation, several nudges are used in the present study. A nudge coined by Thaler, is a gentle push towards the right direction without banning people’s free choices [50], with the goal of slightly adjusting the subconscious behavior [51]. Further, evidence suggests that people are loss averse (consider losses to be more important than gains), regret averse (regret losses that have been made because of poor decision making), present biased (prioritize short-term gains at the expense of long-term gains) and are unrealistically overconfident (overestimate their abilities) [52].

In the present study, in order to enhance participation retention, a head-start nudge will be used to reach potential participants who can benefit the most of the intervention. In addition, a loss-aversion nudge will be used to exploit loss-aversion bias to promote smoking cessation and adherence to smoking abstinence. This nudge will offer participants a chance to win a prize if they stop smoking and adhere to smoking abstinence. Once committed and not wanting to regret the loss of winning the prize, loss aversion or regret aversion can act as motivational tools to push the participants to comply with not smoking and maintain smoking abstinence.

### Pilot study

Between October 2017 and January 2018, the authors piloted the BeHealthyR intervention in a community center in Rotterdam to test its feasibility. A total of eight participants were recruited and assigned to the SM or SM-B condition. Of the eight, six participants (Mean_age_ = 47.2, SD_age_ = 8.4; 100% women) completed the intervention and filled in all questionnaires at baseline (T0), four weeks after baseline (T1) and twelve weeks after baseline (T2). The results of the pilot study allowed for an evaluation of the primary outcomes (stress, smoking and financial health) and the limitations of the methodology. Mean scores for stress, mean number of cigarettes for smoking and proportions for financial health were calculated (See Methods section). The mean scores for stress decreased from 37.2 (T2) to 21.5 (T0) (*p* > 0.05). The mean number of cigarettes smoked per day decreased from 16.2 (T0) to 15.0 (T2) (*p* > 0.05). No changes were seen in financial health (data not shown). Challenges were identified in the recruitment of participants. Also, there was a need to refine the questionnaires (i.e. to ease understanding of questions on smoking) and the study (i.e. to offer flexibility and support to participants to complete the questionnaires). Further, participants considered it important on being provided with additional strategies to quit smoking and maintain smoking abstinence. For the main study, we therefore decided to integrate an additional smoking cessation meeting between the fourth (T1) and fifth (T2) group sessions. During this meeting, professional smoking cessation counselors will give advice on how to quit smoking, provide self-help materials and information on smoking cessation programs. Lastly, technical and logistic constraints associated with coordination and recruitment of participants between Erasmus University Rotterdam (EUR) and Dutch mental healthcare organizations, Indigo Rijnmond and Avant Sanare were identified and improved.

## Methods/design

### Aims

This paper presents the study protocol for a BCI to improve the health of low-SES residents living in Rotterdam. The main aim of the current study is to evaluate the efficacy of a theory-based multicomponent intervention for low-SES residents living in Rotterdam. We hypothesize that in comparison to those in the control condition, participants receiving the intervention will have lower stress levels, will smoke less and have improved financial and self-perceived health.

### Study design

BeHealthyR is a three-arm RCT with three points in time: baseline (T0), four weeks post-baseline (T1) and twelve weeks post-baseline (T2). Between February 2018 and July 2019, a total of 300 participants will be recruited and randomized either to a stress management program (SM), stress management with a buddy program (SM-B) or a control condition (CC).

### Study setting and population

In collaboration with the municipality of Rotterdam and mental health organizations (indigo Rijnmond and Avant Sanare), the present study will be conducted in community centres in Rotterdam. A total of 300 residents aged 18 years and above will be included if they perceive stress, smoke and have poor financial health. A list of study sites is available on request from a.schop@euc.eur.nl.

### Recruitment of study population

**Fig. 3:**
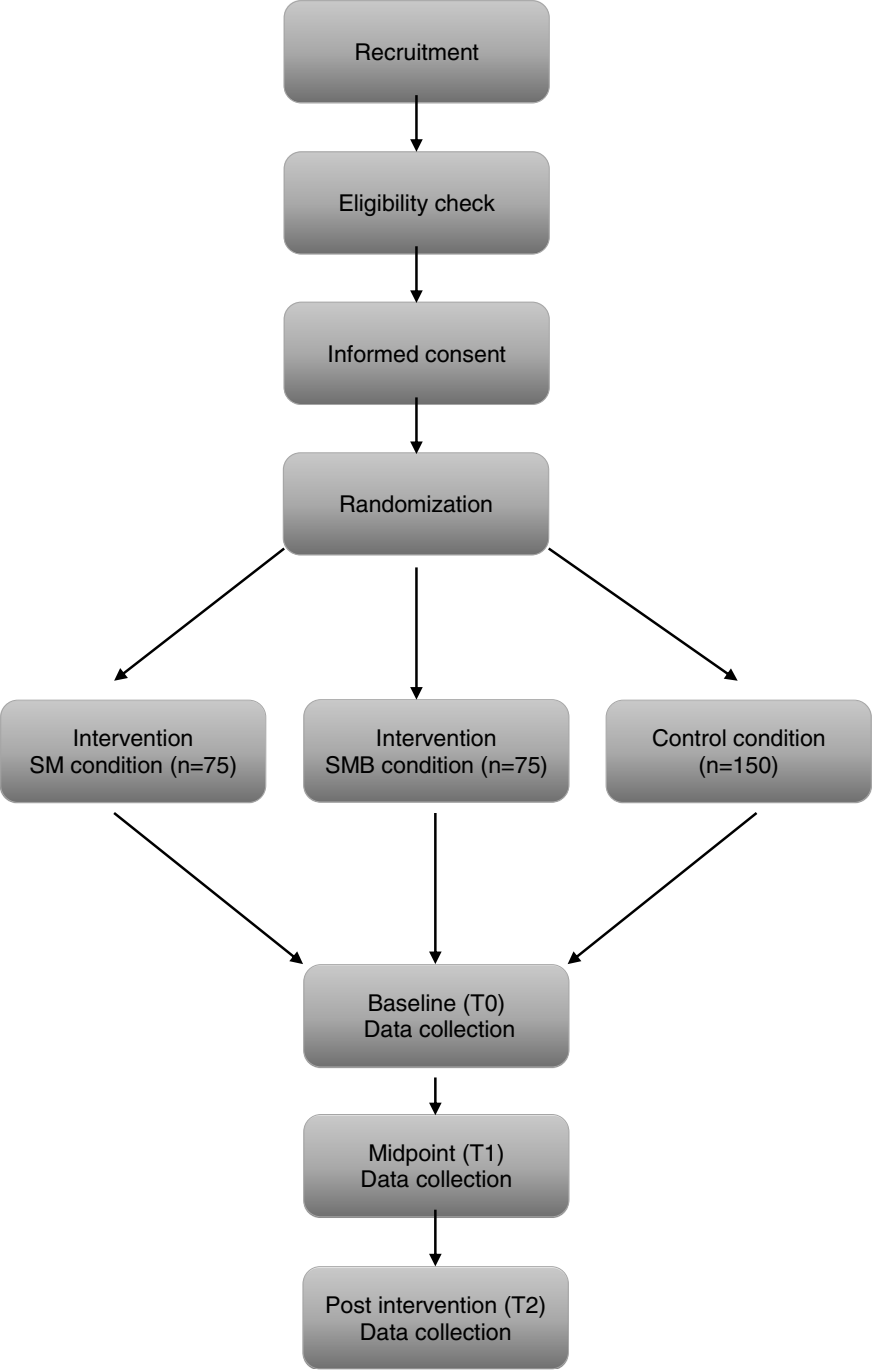
Flowchart of participants. SM = stress management condition; SM-B = stress management + buddy condition; T0 = baseline; T1 = 4 weeks after baseline; T2 = 12 weeks after baseline

Previous research has indicated that a multipoint approach is suitable for recruiting individuals from low-SES as these individuals are difficult to reach out to and less likely to take part in research [21]. Therefore, in the current study, eligible participants will be recruited using a multipoint recruitment strategy which involves recruiting via: 1) information at community and welfare organizations; 2) contacts with active key-figures; 3) online sources (i.e Facebook page and Website), 4) personal phone contacts by Indigo Rijnmond; 5) advertisements in local newspapers; 6) flyers placed in the general public in Rotterdam (i.e. supermarkets); 7) community places (i.e mosques, churches, secondhand shops, daycare centres and food banks); and 8) snowball sampling techniques. Eligible participants who express an interest in participating in the study will be invited to attend an orientation session. During the orientation session, the research team (SS, AS, IB, WV and research assistants) will give presentations about the research study and provide potential participants with information pamphlets containing: 1) an information letter about the study; 2) a screening questionnaire (checking the eligibility criteria) and 3) an informed consent form (see Additional file 1). If a participant meets the eligibility criteria, he/she signs the informed consent and returns it to the research team. A flowchart of participants recruited for the study is shown in Fig. 3.

### Inclusion and exclusion criteria

Eligible participants are over 18 years of age, reside in Rotterdam, perceive stress, smoke and have poor financial health. All study participants must have adequate Dutch-language skills and provide written informed consent to take part in the study. Participants will be excluded from the study if they: 1) follow other stress management or smoking cessation courses; 2) receive help from debt services for their financial problems; or 3) have health problems which hamper their ability to take part in the study.

### Randomization and blinding

**Fig. 4:**
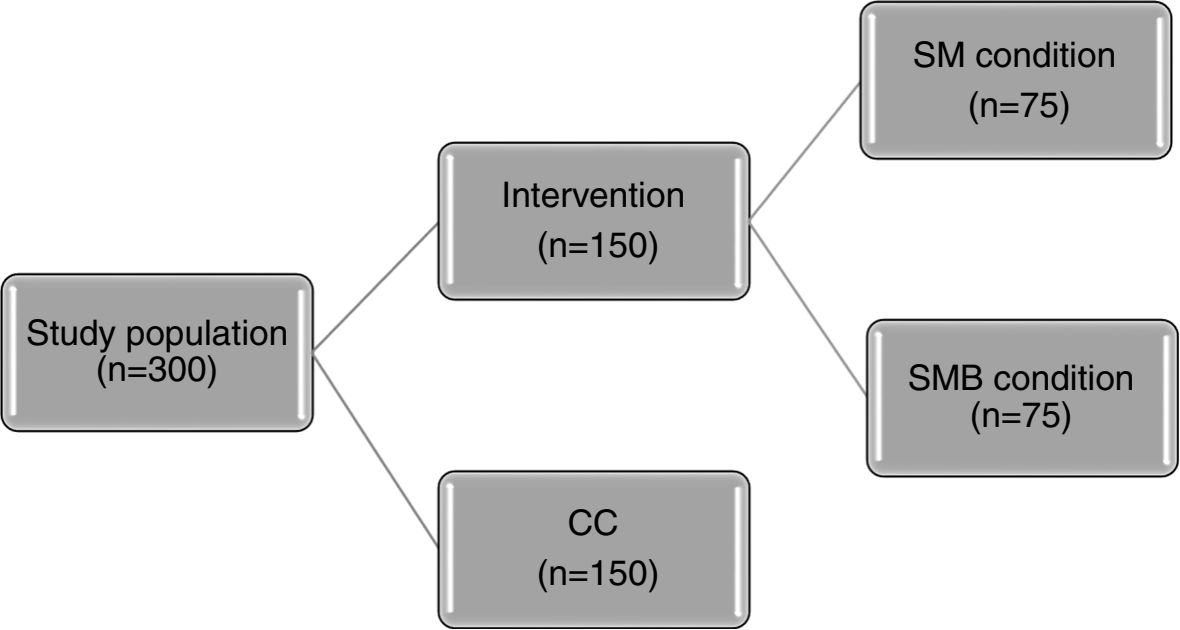
Randomization of study participants. SM = stress management condition; SM-B = Stress management + buddy condition; CC = control condition

Once eligibility is checked and consent is gained, participants will be randomly allocated to three conditions in a 1:1:2 allocation ratio using computer generated random numbers. For practical and logistic reasons (i.e low number of participants per group session), randomization will be done on group level (i.e potential participants attending a specific training session at a community center will be randomized to the same intervention arm). To minimize the risk of bias, randomization will be done by the principal investigator (AS) Fig. 4 shows the randomization scheme of study participants.

Given the nature of the intervention, it is not possible to blind participants or the research team involved in delivering the intervention (IB and WV) and data collection (SS, AS and research assistants). Therefore, to reduce the risk of bias and to ensure that data collection is conducted in accordance with the ethical guidelines, research assistants will receive a training in data collection. Further, AS will maintain regular contact (biweekly meetings) with the research team to ensure that study procedures conducted are consistent with sound research design.

### Intervention and comparison groups

#### Intervention group with stress management course only (SM)

The SM program is an adaption of the existing course *Less Stress* developed by Trimbos institute [53] and has been tailored to meet the specific needs of low-SES participants. A core element of SM is its group-based format in which psycho-educative topics on stress responses and coping and motivation to stop smoking link up with cognitive and behavioral technique activities. The SM consists of four weekly sessions (1.5 h/session) and a follow-up session eight weeks later. The psycho-educative topics are discussed in the following order: introduction to stress, thinking habits, coping skills, setting boundaries, goal-setting and self-affirmation exercises, motivation to stop smoking and self-reflection. In session one, participants are offered general information on stress, the types of stress, stress signals and the effects of stress on health. After some theoretical background, participants learn the techniques of progressive relaxation and breathing exercises. Furthermore, BCT is utilized in the form of a 20- min goal-setting activity in which participants will be asked to set goals. This activity will be repeated once every month over the course of the study duration. In session two, BCT is utilized in the form of a 20-min self-affirmation activity carried out during the session. To increase self-affirmation, participants will be asked to write about their core values (i.e family, religion), why these values might be important to them and to rank these values in terms of their personal importance. The self-affirmation task is performed once over the course of the study period. Also, in session two, cognitive techniques (CT) are applied in which participants will be asked to identify, reframe and replace negative thinking habits with positive ones. In session three, participants will learn how to apply coping skills learned in previous sessions. In session four, participants will learn about healthier habits on how to reduce physical stress reaction and also rehearse the knowledge and techniques learned in previous sessions. Between sessions four and five, participants will be invited to a smoking cessation meeting during which professional smoking cessation counselors will provide information on strategies on how to stop smoking and maintain smoking abstinence. Participants will also receive self-help material and information on smoking cessation programs. Finally, in session five, participants will be asked to create their own Relapse Prevention Map (RPM). By creating a personalized RPM, participants will be able to plan ahead for high risk situations (i.e how to cope better with stress or how to resist smoking cravings).

#### Intervention group stress management with a buddy condition (SM-B)

The stress management with a buddy condition (SM-B) includes the same psycho-educative topics and exercises, CT and BCT activities as the SM condition. The SM-B utilizes one-to-one support through a buddy selected by the participant (the cursus trainer matches a buddy with a participant). A buddy, 18 year or older student or volunteer is recruited and subsequently trained by Indigo Rijnmond. The buddy receives a full training on the structure of the SM course program, his/her role on coaching and supporting a participant to get a grip of his/her personal finances (i.e trough organizing and managing finances). Upon completion of this training, a buddy pairs up with a participant and depending on the needs of the participants and if necessary, provides the following: 1) supports participant in managing and filling in tax/welfare papers; 2) helps a participant to get a grip over his/her finances (i.e by making a financial plan); 3) offers participant to overcome daily barriers (i.e arranging childcare); and 4) helps participant to set up a cost plan (i.e participant can track his/her spending on cigarettes). Over the duration of the course, the buddy meets up six times with a participant every second week in a public area. Depending on the needs and wishes of the participant, the buddy can meet up with a participant for one to two hours up to twelve times. After every appointment with the participant, the buddy reports back on the meetings with the participant, the topics discussed during the meetings and the help offered.

#### Control group

Participants in the CC are instructed to continue with their normal behavior. The CC group will complete the questionnaires and objective measurements at the equivalent times as the intervention groups, thus at T0, T1 and T2. After the control period, participants in the CC will be offered the intervention.

### Nudges

#### Head-start nudge

During the orientation sessions of the present study, eligible participants will receive a head-start nudge in the form of a stamp card. On a group-level, participants will be randomly assigned to either receive a half-filled stamp card (three out of eight boxes are already stamped) or an empty stamp-card. By already having a half-labelled stamp card, a participant will feel that he/she has already achieved ‘something’ and this thinking will further motivate the participant to complete the stamp card. Each time a participant attends a group session, he/she gets a stamp. In case a participant misses a session, he/she will receive a stamp only if he/she can provide a good reason (i.e no care available for a sick child). By not penalizing a participant for a missed session (given a good reason is provided), the participant will feel connected to the program and know that his/her presence was missed. This will motivate the participant to avoid missing any session in the future. A full stamp card means the participant will be rewarded with a full bag of groceries after completing the intervention.

#### Loss-aversion nudge

During the first group session, participants will be told that they will receive a ‘surprise’ prize (20€ voucher). The participants will be told that they can only claim the prize if they have attended all five group sessions, and if they have adhered to smoking abstinence. If participants have attended all five group sessions and their smoking abstinence is biochemically verified at T2, they will be awarded with this 20€ reward.

### Retention of participants

**Fig. 5:**
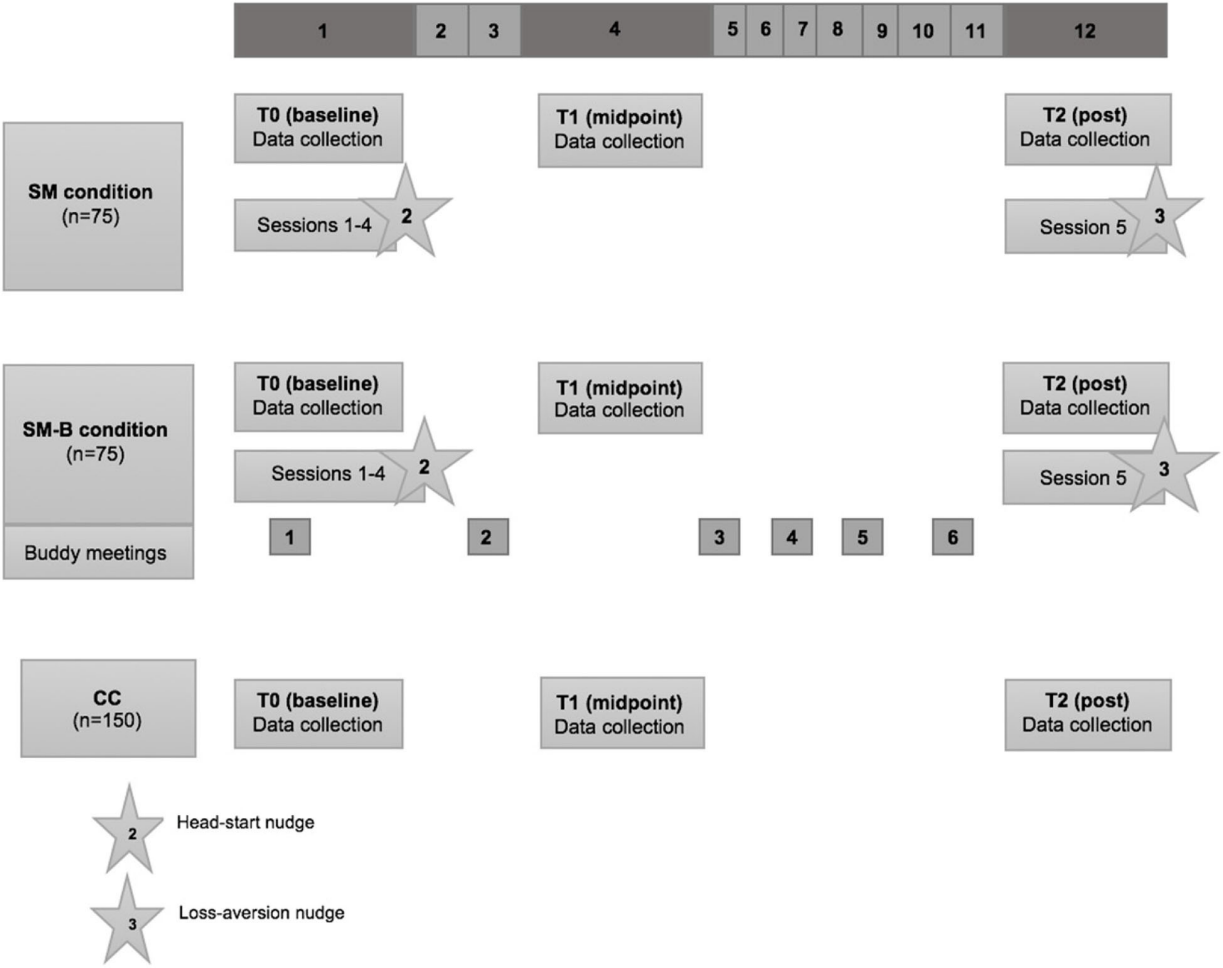
Study timeline. SM = stress management condition; SM-B = stress management + buddy condition; CC = control condition; T0 = data collected at baseline; T1 = date collected 4 weeks after baseline; T2 = data collected 12 weeks after baseline

In order to maintain good participant retention, participants in the SM, SM-B and CC conditions will be rewarded with a gift voucher for their participation and completion of questionnaires at T0 (10€), T1 (10€), and T2 (5€). In Fig. 5, a schematic overview of the intervention and its components is given.

### Data collection methods

#### Outcome measures

The time schedule of study enrollment, interventions and assessments according to the SPIRT guidelines [54] can be found in Table 1.

**Table 1:**
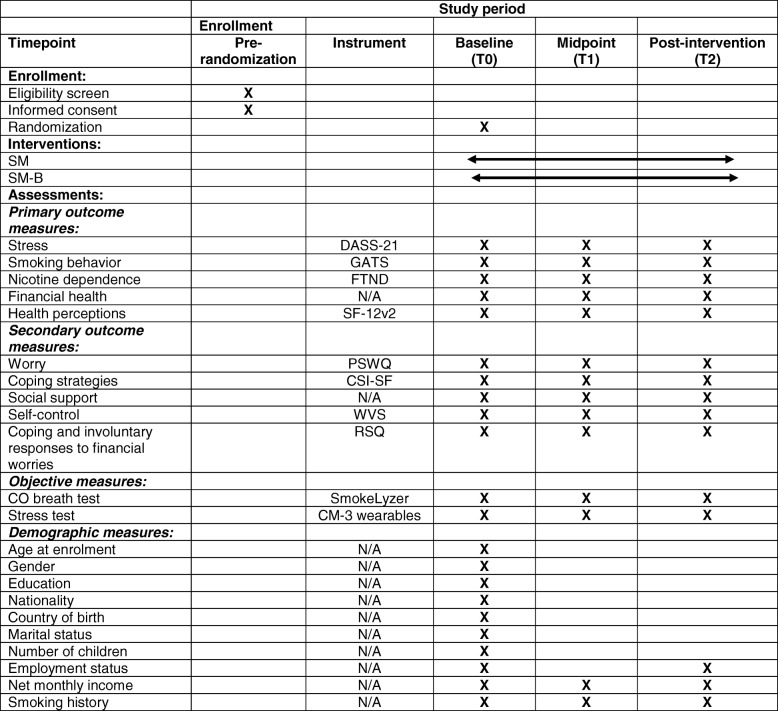
Timetable of study enrollment, interventions and assessments

*DASS-21* Depression, Anxiety and Stress Scales, *GATS* Global Adult tobacco Survey, *FTND* Fagerstrom test for Nicotine Dependence, *SM* Stress management, *SM-B* Stress management + buddy, *SF-12v2* Short Form Health Survey version 2, *PSWQ* Penn state worry questionnaire, *CSI-SF* Coping strategy inventory short form, *WVS* World Value Survey, *RSQ* Responses to stress questionnaire, *N/A* Not applicable

### Primary outcomes

#### Depression, anxiety and stress

The Depression, Anxiety and Stress Scales (DASS-21) is a validated self-report tool consisting of three 7-item measure assessing depression, anxiety and stress over the previous week using a 4-point Likert scale ranging from 0 to 3 (never, sometimes, often and almost always) [55]. Each subscale has a possible range of scores from 0 to 21 with higher scores indicating higher levels of depression, anxiety, and stress [55]. The total scores of each subscale is summed and multiplied by two to yield equivalent scores to the full DASS-42. The DASS-21 is a reliable screening tool for symptoms of depression, anxiety and stress, has been extensively utilized in a large non-clinical population [56] and has internal consistency and concurrent validity in acceptable to excellent ranges (α for depression, anxiety and stress: 0.94, 0.87 and 0.91, respectively) [57].

#### Smoking behavior and nicotine dependence

The Global Adult Tobacco Survey (GATS) questionnaire is used to record participant’s smoking status [58]. Participants will be asked to report the following: 1) current tobacco smoking status, 2) number of tobacco products smoked per day, 3) average tobacco products smoked per day, 4) frequency of consumption of different tobacco products, and 5) motivation to attempt quit. Additionally, participants will be asked to fill out the Dutch version of the Fagerstrom Test for Nicotine Dependence (FTND), which is a 6-item measure assessing smoking habit and dependence [59]. The items of the FTND are summed to yield a total score ranging from 0 to 10 with higher scores reflecting greater dependence. The Dutch FTND has demonstrated acceptable reliability (a = 0.66-0.71) [59] and correlates significantly with number of cigarettes smoked per day [60].

#### Financial health

To measure financial health, participants will be asked to report: 1) their total monthly income (< 1750€, 1750-2750€, > 2750€, don’t want to report); 2) whether they receive welfare (yes, no); 3) their spouse/partner’s total monthly income (don’t have any partner, < 1750€, 1750-2750€, > 2750€, don’t want to report, I don’t know); and 3) whether they have experienced money shortages in the past month (1 = never, 2 = sometimes, 3 = frequently, 4 = often and 5 = almost always). The proportion of participants in each category for each question asked will be calculated.

#### General health perceptions

The Short Form Health Survey version 2 (SF-12v2), is 12-item with three dimensions both for functioning (physical, social and role) and for wellbeing (mental health, general health perceptions and pain) [61]. In this study, participants will be asked to rate their general health on a Likert-type scale (1 = poor, 2 = fair, 3 = good, 4 = very good, and 5 = excellent). Low scores (poor) on the general health scale represent a person who believes his/her health to be poor and high score (excellent) represents someone who sees his/her health as excellent. The SF-12v2 has demonstrated relatively high reliability and validity estimates for the general population [61].

### Secondary outcomes (psychometric measures)

#### Worrying

The Penn State Worry questionnaire (PSWQ), a 3-item questionnaire is designed to measure worry (i.e. many situations make me worry). Participant responses on each item is rated on a 4-point Likert type scale (1 = never, 2 = sometimes, 3 = often and 4 = always). Subsequently item scores are summed up to yield a total score ranging from 0 to 15 with higher scores indicating greater tendency to worry [62]. The 3-item version of the PSWQ is chosen because it can be quickly administered, scored, have psychometric properties similar to the standard 16-item version [62] and has been reported to have an acceptable internal consistence reliability as evidenced by Cronbach’s α = 0.85 [62].

#### Coping strategies

The coping strategy inventory short form (CSI-SF), a 16-item self-report questionnaire derived from an original 78-item CSI [63], will be used to assess coping thoughts and behaviors in response to a specific stressor [64]. In the current study, participants will be asked to rate their coping strategies when confronted with a stressor on a 5-point Likert scale (1 = never, 2 = seldom, 3 = sometimes, 4 = always and 5 = almost always). Participants will receive scores for each first-tier subscale (engagement and disengagement) with scores ranging from 8 to 40. Further, for each of the four item subscales (problem-focused engagement, emotion-focused engagement, problem-focused disengagement and emotion-focused disengagement), participants will receive scores ranging from four (least adherent) to 20 (most adherent) [64]. Higher scores on the problem-focused engagement subscale indicates the participant is actively coping with a particular stressor, while higher scores on the emotion-focused disengagement subscale means the participant is coping negatively with a particular stressor. The CSI-SF is chosen because it contains 16-items, is quick and has been shown to be reliable for measuring coping strategies [64]. Good internal consistency of each coping scale assessed via Cronbach’s alpha (α = 0.58–0.72 [41] and (α = 0.56–0.80) have been demonstrated [65].

#### Social support

Social support will be measured based on a single item by asking participants to list the number of people in response to the question ‘how many people can you turn to for support when experiencing stress?’

#### Self-control

Using the World Values Survey questionnaire (WVS) [66], self-control will be measured using an item where participants will be asked how much freedom of choice and control they have over their lives (1 = no control, 10 = full control). Lower levels mean individuals have no control over their lives, whereas higher levels mean individuals have full control over their lives.

#### Coping and involuntary responses to financial worries

The Responses to Stress Questionnaire (RSQ) is designed to capture the way individuals cope with and react and adapt to stressors [67]. In the current study, the stressor is financial worries (money troubles). Using a single-item, participants will be asked to rate having control over their finances on a 10-point Likert-type scale (1 = no control, 10 = full control). Lower values represent no control and higher values represent full control. The RSQ has been previously tested for different stressors experienced by different populations (children and adults) and have demonstrated strong reliability and validity [67, 68].

### Objective measures

#### Carbon monoxide breath test

Carbon monoxide (CO) measured in parts per million (ppm) and carboxyhemoglobin (COHb) measured as percentage of oxygen replaced) are indicators of smoking status. CO_breath_ and COHb levels will be measured using the Micro+ Smokerlyzer® (Bedfont Scientific Ltd.) by a trained research staff at T0, T1, and T2. Briefly, participants will be asked to hold their breath for 15 s and subsequently to exhale slowly and fully into the mouthpiece of the instrument during which CO_breath_ is recorded. If a participant is not able to hold his/her breath for 15 s, he/she is allowed to hold his/her breath as long as possible and subsequently exhale in the SmokerLyzer. CO_breath_ levels are defined as equivalent to nonsmoker (0-6 ppm), borderline smoker (7-9), light smoker (10-15 ppm), moderate smoker (16-25 ppm), heavy smoker (26-35 ppm) and very heavy smoker (36+) [69]. In this study, a cut-off value of 10 ppm will be used to discriminate smokers from nonsmokers. This value is programmed into the SmokeLyzer device [69] and equals that recommended by other studies [70, 71]. Prior to use, the device is calibrated according to the manufacturer’s instructions and will be re-calibrated again during data collection as an additional measure to ensure accuracy of results. The Micro+ Smokerlyzer® is non-invasive, easy to use, has a precision of < 2% [69] and has been validated in other populations including low-SES [72].

#### Stress test based on heart rate variability data

Previous studies have shown that heart rate variability (HRV) is the most reliable indicator of stress [73, 74]. In the current study, HRV is measured using a wrist wearable sensor equipped with the Philips Cardio and Motion Monitoring Module (CM3-Generation-3, Wearable Sensing Technologies, Philips, Netherlands) which is an integrated module equipped with a photoplethysmographic (PPG) and an accelerometer sensor. Briefly, the wearable is mounted on a participant’s wrist and heart rate is recorded for five minutes in a resting state. After five minutes, the wearable is unmounted and the recorded data is extracted and used for HRV analysis. Compared to on-site clinical monitoring, wrist wearables are efficient, cost-effective, non-invasive, user-friendly, and have been previously validated [75].

### Data management, monitoring and safety

All study materials have been drafted to ensure accurate data collection. All data collected by the research team using paper questionnaires will be kept in locked cabinets at the Erasmus University College (EUC). Data collected from the BeHealthyR website will be downloaded by SS. For confidentiality purposes, all data will be anonymized by the means of an identification number and will be housed on the EUR document vault, a secure digital environment which acts compliant to the General Data Protection Regulation (GDPR). Data backup will be made regularly to ensure data security. Only SS and AS will have access to the collected data. All data will be kept for 10 years after the completion of the study as suggested by the Medical Ethic Review Committee (METC) of the Erasmus Medical University (EMC). Because of the low-risk nature of the BeHealthyR intervention, no adverse events are expected. Therefore, no Data Monitoring and Ethics Committee (DMEC) and interim analyses are required. In case of adverse event, participant complaints or a breach of confidentiality, AS will report the events to the METC according to the ethical guidelines.

### Statistical analysis

#### Sample size calculations

Sample size calculations are based on the primary outcome measures (stress, smoking, and perceived health). To the best of our knowledge, no previous studies have evaluated a similar intervention in low-SES groups. Therefore, it is challenging to determine a prior the necessary sample size for multicomponent behavioral interventions. Previous studies that have evaluated similar components on other outcome measures recommend to aim for a sample size of 20-500 participants per intervention condition with an alpha error of 0.05 and a power of 0.80. to detect the expected effect. Assuming expected attrition of 10%, an initial sample of 300 participants are needed, thus 150 in each intervention condition. All power calculations are done using G*Power [76].

### Data analysis

All statistical analyses will be performed using the Statistical Package for the Social Sciences (SPSS v21) software program. Baseline descriptive statistics will be conducted to describe the study sample using two-sided t-test for continuous variables (i.e. age at enrolment), chi-square tests or Fisher’s exact tests for categorical variables (i.e. gender, nationality, level of education). Logistic regression analysis will be performed for outcomes with two categories (i.e. good health vs bad health, high stress vs low stress, intention to quit vs no intention to quit). Multinomial regression analysis will be conducted for outcomes with more than two categories (i.e. improved, constant or deteriorating financial situation). Age, gender, and nationality will be treated as potential confounding variables. Missing data will be evaluated (depending on the amount and pattern of missingness) using different approaches. Sensitivity analyses will be done using imputation techniques for missing data to assess the robustness of the main analysis.

### Process evaluation and cost-effectiveness

To determine the feasibility of the intervention and to identify factors that may influence its effectiveness, a process evaluation will be conducted. The Medical Research Council (MRC) guidelines for process evaluation of complex interventions [77] will be used to examine implementation (e.g. dose, fidelity), mechanisms of impact, and effect of context on implementation and outcomes. Process data will be gathered via analyses of documents, and qualitative interviews with trainers and study participants. Assuming that the intervention will be effective, a cost-effectiveness analysis will be performed from both a healthcare and societal perspective [78].

## Discussion

This paper describes the protocol for an RCT designed to evaluate the effectiveness of a multicomponent theory-based intervention. It is hypothesized that after a successful implementation of the intervention, the intervention will lead to a reduction in stress, smoking, and improvement in financial and self-perceived health. Previous studies have raised concern about the lack of effective interventions for low-SES populations [16]. By conducting this study, we hope to fill these evidence gaps. This study has several strengths. A key strength is the originality of the study. To the best of our knowledge no previous studies have evaluated the effects of similar components used in the current study to reduce stress, smoking, improve financial and self-perceived health in low-SES groups. Another strength of the current study is that it addresses the efficacy of the intervention by using an RCT design. Random allocation of study participants to the different conditions excludes all possible bias (selection and allocation bias). Another strength is the follow-up period of 12 weeks which allows insight into short-term effects. Finally, by incorporating a process evaluation and a cost-effectiveness analysis, we can gain insight into the facilitators/barriers of the implementation and whether the intervention can be scaled up in different settings, respectively. Finally, the intervention will be conducted in a real-life setting, which will make to generalize the effects to similar low-SES groups in the Netherlands.

Some limitations should be considered. First nonadherence and drop-outs could occur. Previous studies have indicated that low-SES individuals drop out quickly from preventive interventions [17]. In the current study, we have taken this into account. In order to prevent high dropout and maintain participation retention, we have used nudges, to reward and motivate the study participants to stay engaged in the intervention. In addition, the use of buddies for the financial support can be a risk for professionalism of our interventions because these buddies are unexperienced. However, the risk is minimal because these buddies are selected carefully and receive extensive training on financial debts by trained psychologists. By using trained buddies, the intervention costs will be very low. If proven effective, the intervention can be scaled up to different settings which could be an advantage for its cost-effectivity.

In summary, this study will add to the existing knowledge in that we: 1) focus on improving the health of low-SES individuals by targeting multiple factors; 2) use a theory-based multicomponent intervention instead of a single-component intervention; and 3) conduct the study in a real-life setting among a vulnerable population. Taken together, the findings of the current study will contribute to filling the evidence gaps. If proven effective, the findings from the present study will serve to inform future directions of research and clinical practice with regard to behavioral change interventions for low-SES populations on a national and international level.

### Trial status

The BeHealthyR study is currently ongoing. Data collection will be completed in June 2019.

## Additional files

Additional file 1: Participant informed consent. (DOC 31 kb)

## Abbreviations

BCI: Behavioral change intervention
BCT: Behavioral change techniques
CBSM: Cognitive behavior stress management
CBT: Cognitive behavior therapy
CC: Control condition
CM3: Cardio and Motion Monitoring Module
CO: Carbon monoxide
COHb: Carboxyhemoglobin
CSI-SF: Coping Strategies Inventory Short Form
DASS-21: Depression Anxiety Stress Scale
DMSC: Data Monitoring Steering Committee
EUC: Erasmus University College
EUR: Erasmus University Rotterdam
FTND: Fagerstrom Test for Nicotine Dependence
GATS: Global Adult Tobacco Survey
GDPR: General Data Protection Regulation
HRV: Heart rate variability
MRC: The Medical Research Council
N/A: Not Applicable
PP: Positive psychology
PPG: Photoplethysmographic
PSWQ: Penn State Worry Questionnaire
RCT: Randomized controlled trial
RSQ: Responses to Stress Questionnaire
SCT: Social cognitive theory
SES: Social economic status
SF: Short Form
SM: Stress management condition
SM-B: Stress management with a buddy condition
SPIRIT: Standard Protocol Items Recommendations for Intervention Trials
TMSC: Transactional model of stress and coping
WVS: World Values Survey
