# Supplementary material for: The BeHealthyR Study: a randomized trial of a multicomponent intervention to reduce stress, smoking and improve financial health of low-income residents in Rotterdam
Source: BMC Public Health. 2018 Jul 18;18:891. doi: 10.1186/s12889-018-5728-7 (PMC6052714; doi:10.1186/s12889-018-5728-7)
Supplement: Supplementary file 1 — Participant informed consent. (DOC 31 kb) [file 12889_2018_5728_MOESM1_ESM.doc]

**INFORMED CONSENT FORM**

Title of Research Project: Grip en Gezondheid: An integral approach to reduce stress, smoking and improve financial health of residents in Rotterdam.

Name of Principal Investigator: Dr. Astrid Schop-Etman

I hereby confirm that:

1. I have read the information sheet regarding the research project and I have been able to formulate any question on the project. Also, I have received enough information on the project.
2. I know that that my participation is voluntary and I can withdraw out of the study at any time without any need to justify my decision.
3. I understand that my participation means that I will be following the course *Less Stress* and that I will receive help with financial problems. I understand that I will asked to fill in qustionnaires, take a breathing test and a heart test at three time points: during my initial visit, four weeks after my initial visit and two months after my initial visit.
4. I understand that I have the right not to answer questions if I don’t wish to.
5. I understand that the information provided by me will be held confidentially.
6. I understand that I can contact the principal investigator (Dr. Astrid Schop-Etman), in case of concerns, complaints or questions.
7. I confirm that I have read and understand the participant information sheet for the Grip en Gezondheid study. I have had the opportunity to consider the information, ask questions and have had these answered satisfactorily.
8. I am 18 years or older.

I hereby give my consent to participate in the Grip en Gezondheid research project:

|  |  |  |  |  |
| --- | --- | --- | --- | --- |
| Name of Participant |  | Signature |  | Place and Date |

|  |  |  |  |  |
| --- | --- | --- | --- | --- |
| Name of Principal investigator |  | Signature |  | Place and Date |

*You will receive a copy of the signed informed consent.*
